# Babao Dan decreases hepatocarcinogenesis by inhibiting hepatic progenitor cells malignant transformation via down-regulating toll-like receptor 4

**DOI:** 10.3389/fonc.2023.1073859

**Published:** 2023-05-12

**Authors:** Lei Liang, Lu-Yao Zhang, Wen-Ting Liu, Chen Zong, Lu Gao, Rong Li, Qiu-Dong Zhao, Na-Ping Zhao, Li-Xin Wei, Li Zhang, Zhi-Peng Han

**Affiliations:** ^1^ Tumor Immunology and Gene Therapy Center, Third Affiliated Hospital of Naval Medical University, Shanghai, China; ^2^ Department of Hepatobiliary, Pancreatic and Minimal Invasive Surgery, Zhejiang Provincial People’s Hospital, People’s Hospital of Hangzhou Medical College, Hangzhou, Zhejiang, China; ^3^ Key Laboratory of Tumor Molecular Diagnosis and Individualized Medicine of Zhejiang Province, Hangzhou, China; ^4^ Clinical Research Unit, Changhai Hospital, Naval Medical University, Shanghai, China; ^5^ Department of Clinical Pharmacology, The Second Hospital of Anhui Medical University, Hefei, China; ^6^ School of Pharmacy, Anhui Medical University, Hefei, China

**Keywords:** hepatocellular carcinoma, hepatic progenitor cell, Babao Dan, lipopolysaccharides, toll-like receptor 4

## Abstract

**Background:**

Babao Dan (BBD) is a traditional Chinese medicine that has been widely used as a complementary and alternative medicine to treat chronic liver diseases. In this study, we aimed to observe the effect of BBD on the incidence of diethylnitrosamine (DEN)-initiated hepatocellular carcinoma formation in rats and explored its possible mechanism.

**Methods:**

To verify this hypothesis, BBD was administrated to rats at a dose of 0.5g/kg body weight per two days from the 9th to 12th week in HCC-induced by DEN. Liver injury biomarkers and hepatic inflammatory parameters were evaluated by histopathology as well as serum and hepatic content analysis. We applied immunohistochemical analysis to investigate the expression of CK-19 and SOX-9 in liver tissues. The expression of TLR4 was determined by immunohistochemical, RT-PCR, and western blot analysis. Furthermore, we also detected the efficacy of BBD against primary HPCs neoplastic transformation induced by LPS.

**Results:**

We observed that DEN could induce hepatocarcinogenesis, and BBD could obviously decrease the incidence. The biochemical and histopathological examination results confirmed that BBD could protect against liver injury and decrease inflammatory infiltration. Immunohistochemistry staining results showed that BBD could effectively inhibit the ductal reaction and the expression of TLR4. The results showed that BBD-serumcould obviously inhibit primary HPCs neoplastic transformation induced by regulating the TLR4/Ras/ERK signaling pathway.

**Conclusion:**

In summary, our results indicate that BBD has potential applications in the prevention and treatment of HCC, which may be related to its effect on hepatic progenitor cells malignant transformation via inhibiting the TLR4/Ras/ERK signaling pathway.

## 1 Introduction

Hepatocellular carcinoma (HCC), the most common type of primary liver malignancy with increasing incidence in the last decades, is the second leading cause of cancer-related deaths worldwide (1). Approximately 80% of HCC occurs at the end stage of hepatic fibrosis or cirrhotic, when the liver is chronically damaged over a long period of time, it induces a continuous inflammatory response in the liver, which further progresses to liver fibrosis and cirrhosis (1, 2). Over the past few decades, research in inhibiting the occurrence of HCC has yielded remarkable results, but there is still no effective method to reduce the incidence of HCC in clinical practice.

The concept that HCC is a stem cell-derived disease that originates from liver cancer stem cells (CSCs) has recently gained much attention. It is believed that in certain pathological microenvironments, CSCs are mostly derived from normal stem/progenitor cells (3). Hepatic progenitor cells (HPCs), resided in the canals of Hering. Numerous studies have shown that HPCs are bipotential stem-like cells that are defined through their capacity to differentiate toward the hepatocytes and the biliary epithelial cells (4, 5). In a diseased liver section, HPCs’ activation is described as ‘bile duct proliferation’ or ‘ductular reaction (DR)’ in the histology report (6). Previous studies have demonstrated that the proliferative HPCs or DR is related to HCC initiation and also related to early recurrence, which suggested that HPCs may participate in hepatocarcinogenesis (7–12). In addition, some studies have demonstrated that HPCs can be directly induced neoplastic transformation *in vitro* (13–16). Collectively, these researches indicated that HPCs are the origin of liver cancer, and prohibiting HPCs activation may decrease HCC incidence.

HCC is considered to be a tumor associated with inflammation. Inflammatory cytokines are key players in the tumor microenvironment, and they can promote or inhibit cancer formation and progression. LPS is located in the cytoplasm of Gram-negative bacteria and can be taken up in large amounts in the liver in the presence of increased permeability of the intestinal mucosal barrier in cirrhotic rats or patients (17). Our previous study also found that lipopolysaccharide (LPS) can directly induce tumorigenic transformation of primary HPCs in mice by up regulating Ras signaling pathway (13). LPS can increase the expression of extracellular-related kinase (ERK) phosphorylation (18, 19). Moreover, it has been demonstrated that Ras/ERK signaling pathway play an vital role in tumorigenesis and malignant transformation (20, 21). Importantly, our previous study showed that HPCs have high expression of Toll-like receptor 4 (TLR4), which can respond specifically to bacterial LPS (13). Moreover, a previous study suggested that TLR4 could facilitate tumor cell invasion and migration as a cancer stem cell marker in HCC (22). Subsequently, inhibiting the expression of TLR4 may be one of the strategies to inhibit the initiation and progress of HCC.

Babao Dan (BBD) is a classical traditional Chinese medicine which is a powder containing eight constituents, including natural calculus bovis, pearl, musk, snake gall, radix notoginseng, and so on. BBD has been shown to exert curative function in liver damage and it has been widely used as an alternative medicine to treat chronic liver diseases. Our previous study has demonstrated that BBD could inhibit the expression of TLR4 induced by LPS (23). However, whether it can decrease the incidence of HCC and the potential mechanism is still unknown. In this study, we aimed to observe the effect of BBD on DEN-initiated HCC formation and explored its possible mechanism.

## 2 Materials and methods

### 2.1 Reagents

BBD was obtained from Shanghai Pharmaceuticals Holding Co., Ltd, Shanghai, China. Hydroxyproline Testing Kit (A030-2) was bought form Jiancheng, Nanjing, China. (Dulbecco’s) modified Eagle’s medium (DMEM), fetal bovine serum (FBS), penicillin and streptomycin sulfate were purchased from Invitrogen (Carlsbad, CA, USA). The antibody of CK-19(10712-1-AP, proteintech), SOX-9(ab185230, Abcam), Toll-like receptor 4 (TLR4) (ab22048, Abcam), Ras (67648, CST), ERK (4695, CST), P-ERK (4370, CST) and GAPDH (AP0063, Bioworld) were used for western blotting assay or cellular immunofluorescence staining. The kit of endotoxin enzyme-linked immunosorbent assay (ELISA) test, including LPS (H178), IL-6 (H007), TGF-β1(H034), TNF-α (H052), MCP-1 (H115) were purchased from Jiancheng, Nanjing, China. LPS (L14130) was purchased from Sigma. Diethylinitrosamine (DEN) was obtained from Sigma-Aldrich, St. Louis, MO.

### 2.2 Animal models and treatments

Male Sprague-Dawley (SD) rats (180-200g) were purchased from the Shanghai Experimental Animal Center at the Chinese Academy of Sciences in Shanghai, China. All animals were fed in a pathogen-free animal facility and freedom to food and water.

The rats hepatocarcinogenesis model was induced by intraperitoneal injections of DEN once a week with the dose of 70mg/kg for 12 weeks. BBD dissolved in saline and was gavaged in rats with a dose of 0.5g/kg once every two days from the 9th to 12th week HCC-induced by DEN. For this model, rats were randomly divided into four groups (ten rats in each group) and treated as follows: the normal group (gavaged saline), the BBD group (gavaged BBD), the DEN group (gavaged saline), and the DEN/BBD group (gavaged BBD). The rats were sacrificed after the last day of 12 weeks in DEN-induced model.

### 2.3 Measurement of liver function

The serum levels of rats were collected by centrifugation at 3000g, 4°C for 10 min. Alanine aminotransferase (ALT) and aspartate aminotran sferase (AST) have been regarded as markers of liver injury. The levels of AST and ALT were measured by a biochemical analyzer in all experimental rats.

### 2.4 Histological examination and immunohistological staining

Paraffin-embedded liver samples were cut into 4-μm sections for hematoxylin and eosin (H&E) staining for histopathological examination, Sirius red staining for collagen determination. Image-Pro Plus software version 6.2 (Media Cybernetics Inc., Bethesda, MD, USA) was used to analyze the connective tissues stained with Sirius red. To quantify liver fibrosis, a 100 mg wet liver sample was subjected to acid hydrolysis to determine the amount of hydroxyproline according to the protocol in the Hydroxyproline Assay Kit. The Immunohistochemistry (IHC) analysis was performed to determine CK-19, SOX-9, and TLR4 expression. Paraffin sections used for immunohistochemistry assay were 4-μm thick, and the detailed procedure was performed as previously described (23).

### 2.5 Enzyme-linked immunosorbent assay

Interleukin 6(IL-6) in rat liver was measured by enzyme-linked immunosorbent assay (ELISA). The samples were added to the test wells, followed by IL-6 antibody and Streptavidin-HRP. The wells were then sealed with a sealing membrane and incubated at 37°C for 60 minutes with gentle shaking. Afterwards, the sealing membrane was carefully removed and the liquid drained, discarding the remaining water. Chromogenic solutions A and B were then added to each well and incubated for 10 min at 37°C protected from light. Subsequently, termination solution was added to each well and after 10 minutes the optical density (OD) was measured at 450 nm and the concentration was then calculated from the standard. ELISA was performed to detect the secretion of TGF-β1, TNF-α and MCP-1 according to the manufacturer’s instructions.

### 2.6 Isolation of primary HPCs

As shown in our previous study (24), 2-AAF/PH rat model was employed to isolate primary HPCs. 2-AAF was administered at a dose of 10 mg/kg daily for 4 days. On the fifth day, 70% hepatectomy was performed. 2-AAF was continued to be administered for 1 week. SD rats were anesthetized under sterile conditions. The pre-warmed GBSS (Gey’s balanced salt solution) solution (containing 0.125 mg/ml DnaseI) was instilled into the liver via the portal vein, followed by 0.1% type IV collagenase, and the liver was dissected and placed in 0.06% type IV collagenase solution. After shaking at 37°C for 30 min, the liver was filtered through 100 mesh, placed in 0.01% protease solution, shaken at 37°C for 30 min, centrifuged at 50 × g for 5 min, the supernatant was collected, centrifuged at 350 g for 5 min, and resuspended in DMEM medium. OV6-positive cells were isolated from the hepatocyte suspension using immunomagnetic beads.

### 2.7 Cell culture and treatment

Primary HPCs were incubated in DMEM supplemented with 10% FBS and 1% penicillin/streptomycin, in the condition of 37°C, 5% CO_2_. Then, we treated primary HPCs with 10 μg/mL LPS (Sigma) supplemented with 10% Normal rat portal vein serum (normal-serum, v/v) or 10% BBD-treated rat portal vein serum (BBD-serum, v/v) group for 2 months, respectively, to induce neoplastic transformation. We also treated primary HPCs with 10 μg/mL LPS (Sigma) supplemented with 10% Normal rat portal vein serum (normal-serum, v/v) or 10% BBD-treated rat portal vein serum (BBD-serum, v/v) group for 48h, respectively.

### 2.8 Real-time polymerase chain reaction analysis

To test mRNA expression, total RNA from every frozen rat liver tissue and different group cells was extracted by using TRIZOL (Invitrogen, Carls-bad, CA, USA). Prime Script RT reagent Kit (Takara, Kyoto, Japan) was performed for cDNA synthesis. Relative quantitative PCR 9RT-PCR) was performed using SYBR Green PCR Kit (Applied BI) according to the manufacturer instructions. Real-time PCR was performed in a total reaction volume of 10 μl (5 μl SYBR green, 0.5 μl forward and reverse specific primers, respectively, 1 μl complementary DNA and 3 μl ddH2O). PCR conditions used were: 95°C for 10 min, followed by 40 cycles of 95°C for 15 s, 60°C for 30 s, and 72°C for 30 s. The quantitative analysis of relative mRNA was conducted with β –actin as the reference. Primer sequences are listed in **Table 1** as follows.

**Table 1:** The primer sequences of relative genes.

Gene	Forward (5’-3’)	Reverse (5’-3’)
*IL-6*	CCGGAGAGGAGACTTCACAG	ACAGTGCATCATCGCTGTTC
*TGF-β1*	ATACGCCTGAGTGGCTGTCT	TGGGACTGATCCCATTGATT
*TNF-*α	AGATGTGGAACTGGCAGAGG	CCCATTTGGGAACTTCTCCT
*MCP-1*	ATGCAGTTAATGCCCCACTC	TTCCTTATTGGGGTCAGCAC
α *–SMA*	ACTGGGACGACATGGAAAAG	CATCTCCAGAGTCCAGCACA
*Collagen*I	AGGCATAAAGGGTCATCGTG	ACCGTTGAGTCCATCTTTGC
*TLR4*	TGCTCAGACATGGCAGTTTC	TCAAGGCTTTTCCATCCAAC
*β –actin*	GCCAACACAGTGCTGTCTGG	TGATCCACATCTGCTGGAAGG

### 2.9 Western blotting assay

The total protein samples were collected from the fresh liver tissue and treated cells, respectively. The liver tissue and cells were washed with PBS and lysed by RIPA and PMSF (100:1, v/v). After quantification by bicinchoninic acid (BCA) protein assay kits, 20-25 μg protein was separated by SDS-PAGE and transferred to polyvinylidene fluoride membrane. After blocking in 5% fat-free milk/1 × TBS/0.1% Tween-20 for 1 h at room temperature, the membranes were then incubated with diluted primary antibodies overnight at 4°C. Thereafter, the membranes were further incubated with the secondary antibody according to the primary antibody for 1 h at room temperature, and then were visualized using the BeyoECL Plus substrate system (Beyotime).

### 2.10 Cellular immunofluorescence staining

Different group cells were washed with PBS, and then fixed with 4% paraformaldehyde solution and 0.5% Triton X-100 solution in PBS (v/v) at 4°C for 30 minutes. After blocking with 1% bovine serum albumin (BSA), the cells were washed with PBS and incubated with antibodies TLR4 overnight at 4°C, followed by Alexa Fluor 488 goat anti-mouse and anti-rabbit IgG secondary antibodies (37°C for 2h). Subsequently, nucleus was stained with DAPI and observed by a fluorescence microscope.

### 2.11 Colony formation assay

The Colony-forming assay was performed according to previous report (25). For the colony-forming assay, the rat primary HPCs after different treatments for 72h were seeded at a density of 500 cells/well into 6-well plates in standard medium. Colonies formed were fixed with 4% paraformaldehyde, stained with 0.5% (w/v) crystal violet prepared in 0.6% (v/v) glutaraldehyde solution, and counted.

### 2.12 Sphere formation assay

For the Sphere-formation assay, the rat primary HPCs after different treatments were seeded at a density of 5000 cells/dish into 6-cm dishes coated with 1% agarose and incubated for 3-7 days. The diameters and numbers of the sphere were measured under a microscope every 2 days.

### 2.13 Xenografts model

To investigate the effect of BBD on primary HPCs neoplastic transformation induced by LPS. We subcutaneously implanted primary HPCs xenografts in nude mice. 1x10^6^ normal-serum cultured primary HPCs cells, normal-serum cultured primary HPCs with LPS-insulted and BBD-serum cultured primary HPCs with LPS-insulted were transplanted into nude mice (6-8 weeks, weight 20-23g), respectively. After 3 weeks, tumors were detected in all mice.

### 2.14 Statistical analysis

All experiments were performed at least three times. The results are presented as the mean ± standard deviation. Analysis of the differences in hepatic function indicator, hydroxyproline content, and gene expression levels was done by GraphPad Prism 8 (GraphPad Software Inc., CA). Significance between groups was performed using the two-sided independent Student’s t test. P-values less than 0.05 considered statistically significant.

## 3 Results

### 3.1 BBD decreased the incidence of hepatocarcinogenesis

**Figure 1:**
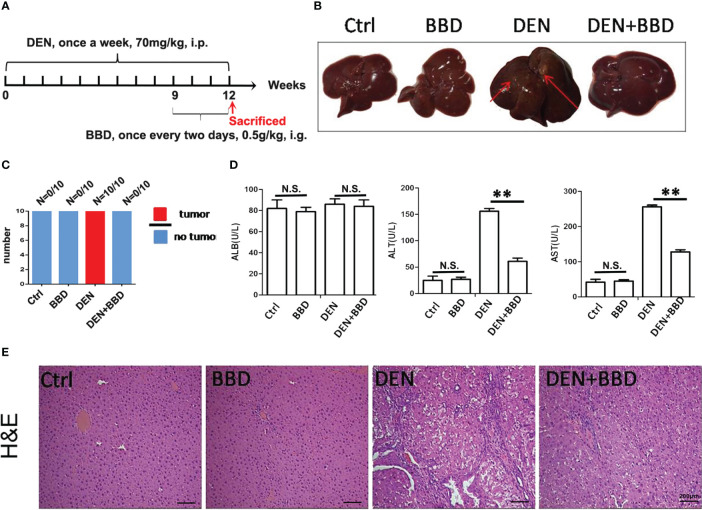
BBD decreased diethylnitrosamine-initiated hepatocellular carcinoma formation and ameliorated liver injury in rats. The rats’ hepatocarcinogenesis model was induced by intraperitoneal injections of DEN at a dose of 70 mg/kg body weight once a week for 12 weeks. BBD was administered to rats at a dose of 0.5g/kg body weight every two days from the 9^th^ to the 12^th^ week. All rats were sacrificed on the last day of 12 weeks. **(A)** Schematic representation of induction of experimental HCC in rats and administration. **(B)** Representative upper and visceral views of rat’s liver in different groups. **(C)** The number of rats with liver cancer in different groups. **(D)** The Serum levels of ALB(U/L), ALT(U/L) and AST(U/L). **(E)** HE staining of liver tissues. Differences were analyzed using a two-tailed Student’s t-test (Compared to DEN group, **P <0.01; compared to Normal group, N.S., no significance).

Firstly, we established a DEN-initiated HCC formation model in rats. DEN was given to rats by intraperitoneal injections once a week at 70 mg/kg body weight for 12 weeks. At the same time, BBD was gavaged to rats at a dosage of 0.5g/kg body weight once every two days from the 9th to 12th week in HCC-induced by DEN. After 12 weeks, all rats were sacrificed (**Figure 1A**). We observed that DEN could induce hepatocarcinogenesis in all rat livers. While, BBD significantly decreased the incidence of HCC (**Figures 1B, C**). The results indicated that BBD could prevent or postpone the initiation of HCC formation. The data suggested that BBD may be a potential novel therapeutic choice for preventing the initiation of HCC.

### 3.2 BBD alleviated liver injury and inflammatory cytokines secretion

**Figure 2:**
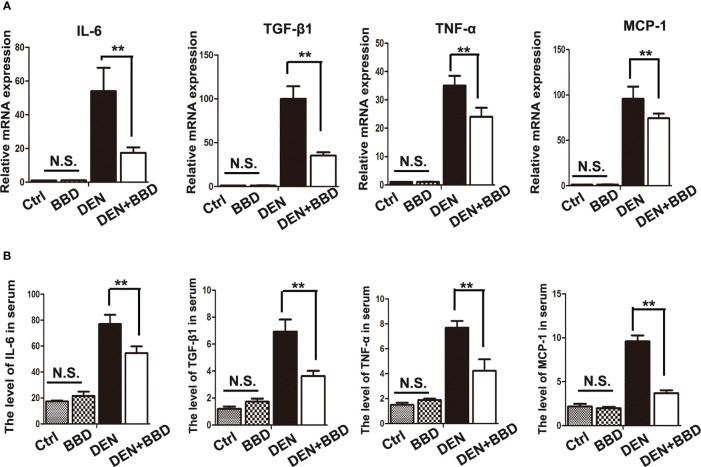
BBD alleviated the secretion of inflammatory cytokines. **(A)** RT-PCR analysis of IL-6, TGF-β1, TNF-α, MCP-1 mRNA in fresh liver tissues. **(B)** ELASA analysis of the level of inflammatory cytokines in peripheral vein serum. The unit is ng/L. Differences were analyzed using a two-tailed Student’s t-test (Compared to DEN group, **P < 0.01; compared to Normal group, N.S., no significance).

Then, we examined the effect of BBD on damaged liver. Serum albumin (ALB), alanine aminotransferase (ALT), and aspartate aminotransferase (AST) were used as indexes to assess liver damage in all experimental rats and measured by biochemical analyzer. There was no difference in the level of ALB. The results also showed that BBD could significantly reduce liver injure compared to DEN group (**Figure 1D**). In addition, we evaluated the potential effect of BBD on inflammatory infiltration in the liver. Liver paraffin sections stained by H&E showed that liver of rats with severe DEN injury and BBD significantly reduced hepatocyte injury and inflammatory cell infiltration (**Figure 1E**). Meanwhile, the expression of inflammatory cytokines, including interleukin 6 (IL-6), transforming growth factor β1 (TGF-β1), tumor necrosis factor α (TNF-α), and monocyte chemotactic protein 1 (MCP-1) were detected by RT-PCR in fresh liver tissues (**Figure 2A**). Moreover, the relevant inflammatory cytokines were also detected in peripheral vein blood by ELISA. The results of RT-PCR and ELISA indicated that BBD significantly reduced the expression of inflammatory cytokines compared with the DEN group (**Figure 2B**). Thus, these results suggested that BBD could protect hepatocytes from DEN damage and inhibit inflammatory infiltration and expression of inflammatory cytokines.

### 3.3 BBD attenuated ductular reaction

**Figure 3:**
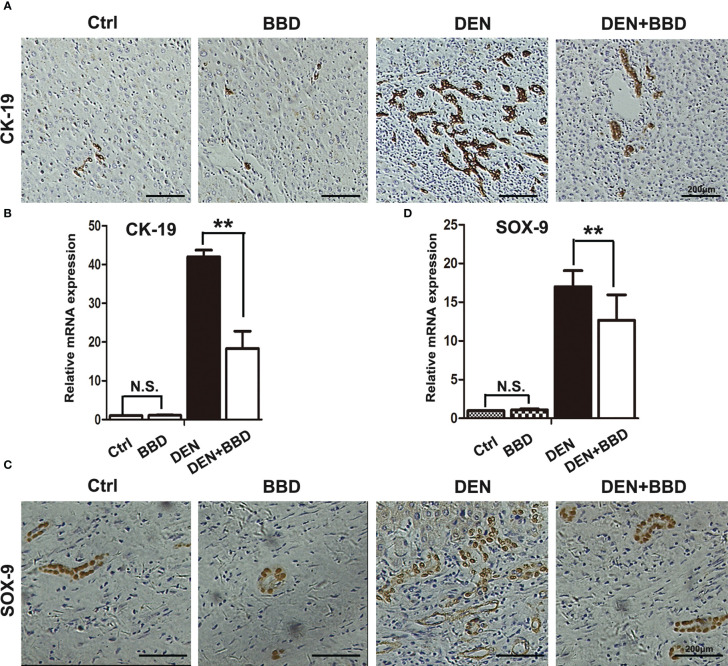
BBD attenuated ductal reaction. **(A, B)** Representative IHC staining of liver sections for the protein expression of CK-19, a marker of HPCs, was analyzed and quantitatively measured by RT-PCR. **(C, D)** Representative IHC staining of liver sections for the protein expression of Sox-9, a marker of HPCs, was analyzed and quantitatively measured by RT-PCR. Differences were analyzed using a two-tailed Student’s t-test (Compared to DEN group, **P < 0.01; compared to Normal group, N.S., no significance).

Evidence had demonstrated that dysregulated HPCs possess tumor-initiating ability *in vivo*. HPCs activation in a diseased liver section is described as ‘ductular reaction’ or ‘bile duct proliferation’ in a histology report. Prevent the activation of HPCs may be an effective method to decrease the incidence of HCC. As a result, we detected the activation of HPCs by immunohistochemical examination of CK-19 (**Figures 3A, B**) and SOX-9 (**Figures 3C, D**). The results showed that DEN could obviously rise the activation of HPCs or ductular reaction, While, BBD ameliorated it.

### 3.4 BBD decrease the expression of TLR4

**Figure 4:**
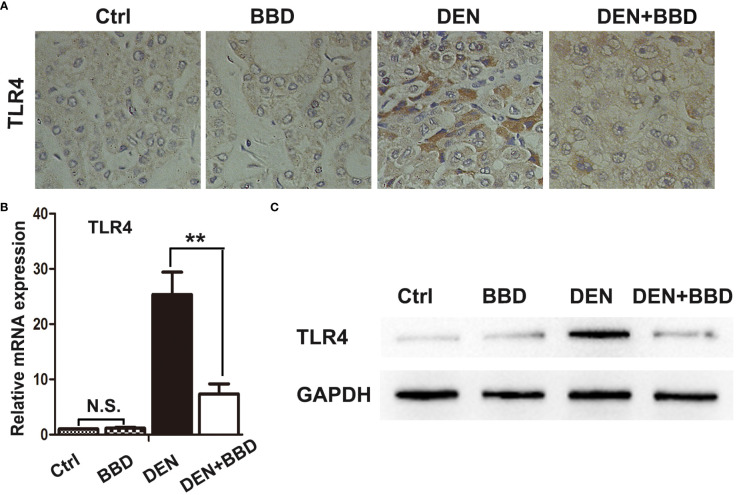
BBD reduced the expression of TLR4. The expression of TLR4, a receptor specially bind to LPS, was determined by IHC-P **(A)**, and quantitatively measured by RT-PCR **(B)** and Western blotting **(C)** assays. Differences were analyzed using a two-tailed Student’s t-test (Compared to DEN group, **P < 0.01; compared to Normal group, N.S., no significance).

Previous study suggested that TLR4 could facilitate tumor cells invasion and migration as a cancer stem cell marker in HCC (22). Moreover, Li et al. has demonstrated that TLR4 highly expressed in HPCs, and LPS could induce HPCs neoplastic transformation via TLR4 pathway (13). So, we examined the expression of TLR4 in rat livers by immunohistochemical examination (**Figure 4A**). At the same time, the expression of TLR4 was also analyzed by RT-PCR and Western blot, accordingly (**Figures 4B, C**). As the figures indicated, compare to DEN group, BBD could obviously down-regulate the expression of TLR4. Collectively, BBD may inhibit HPCs neoplastic transformation by preventing the expression of TLR4.

### 3.5 BBD inhibited rat primary hepatic progenitor cells neoplastic transformation via TLR4/Ras/ERK pathway

**Figure 5:**
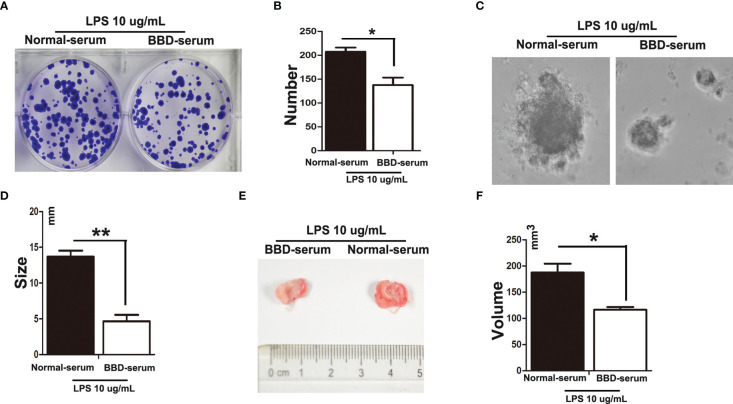
BBD inhibited primary HPCs neoplastic transformation. Primary HPCs were treated with 10 μg/mL LPS supplemented with 10% normal-serum(v/v), or 10% BBD-serum groups for 2 months, respectively, to induce them to undergo neoplastic transformation. **(A)** Representative images of colony formation in 6-well plates in standard medium with different treated primary HPCs for 72h. **(B)** Quantification of Colonies number. **(C)** Representative images of Different treated primary HPCs cells were suspended in serum free medium were seeded and incubated for 3-7 days. **(D)** The size of sphere was observed manually under microscope. **(E)** LPS-induced primary HPCs cells treated with different serum were subcutaneously transplanted into nude mice. **(F)** The volume was compared. Differences were analyzed using a two-tailed Student’s t-test (Compared to normal-serum with LPS group, *P < 0.05, **P < 0.01).

To further explore the effect of BBD on HPCs neoplastic transformation. We isolated and cultured rat primary HPCs *in vitro*. We treated primary HPCs with 10 μg/mL LPS (Sigma) supplemented with 10% normal-serum(v/v), or 10% BBD-serum group for 2 months, respectively, to induce them neoplastic transformation. Subsequently, we detected the influence of BBD on primary HPCs insulted with LPS by colony-formation assay, sphere-formation assay and Cell counting examination (**Figures 5A–D**). The results showed that long-term treatment with LPS could induce primary HPCs neoplastic transformation with high colony-formation, sphere-formation and proliferation. More importantly, cultured with BBD-serum obviously decreased the ability of colony-formation, sphere-formation and proliferation. In addition, 1x10^6^ normal-serum cultured primary HPCs, normal-serum cultured primary HPCs with LPS-insulted and BBD-serum cultured primary HPCs with LPS-insulted were transplanted into nude mice (6-8 weeks, weight 20-23 g), respectively. After 3 weeks, tumors were detected in all mice. As the figure showed, LPS could induce tumor formation, while BBD could significantly inhibit the ability of tumor formation in nude mice (**Figures 5E, F**).

**Figure 6:**
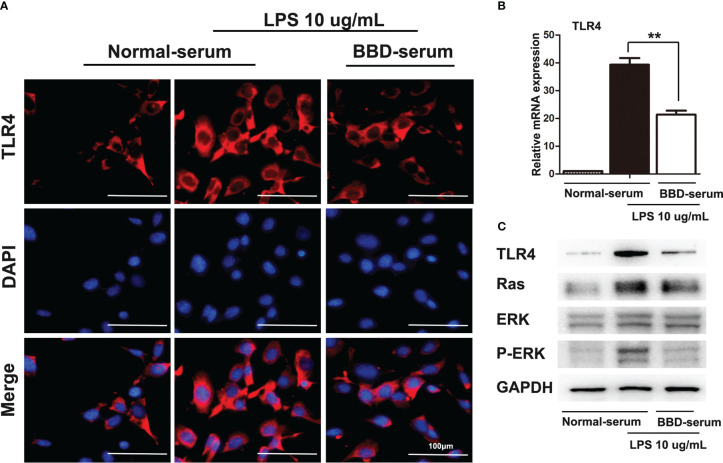
BBD inhibited the LPS-induced HPCs proliferation and malignant transformation by the TLR4/Ras/ERK signaling pathway. **(A)** Cellular immunofluorescence staining was performed to detect the expression of TLR4. LPS was used to induce the expression of TLR4. **(B)** The mRNA expression of TLR4 in cells was quantitatively measured by RT-PCR assay. **(C)** The western blotting assay was used to detect TLR4, Ras, ERK, p-ERK, and GAPDH expressed in LPS-induced HPCs after treatment with BBD-serum at 48 (h) Differences were analyzed using a two-tailed Student’s t-test (Compared to normal-serum with LPS group, **P < 0.01).

Furthermore, cellular immunoflurescence staining was performed to detect the expression of TLR4 (**Figure 6A**). RT-PCR and western blotting assays were performed to detect the expression of TLR4 (**Figures 6B, C**). As the data showed, BBD could significantly inhibit the expression of TLR4. Previous study has demonstrated that LPS pretreated could significantly upregulated the Ras signaling pathway in HPCs (24). And Ras/ERK is considered an important signaling pathway related to tumorigenesis and differentiation. To further demonstrate the mechanism of BBD-serum on HPCs proliferation and neoplastic transformation, the Ras/ERK signaling pathway was analyzed. LPS can up-regulate the expression of Ras and p-ERK via TLR4 signaling pathway in primary HPCs. Then we cultivate primary HPCs and treated LPS meanwhile BBD-serum was administrated for 48h. The results shown that BBD-serum markedly inhibited the expression of Ras and p-ERK in HPCs at 48h. These results suggested that BBD inhibited primary HPCs neoplastic transformation may via preventing the TLR4/Ras/ERK signaling pathway.

## 4 Discussion

In the present study, we showed the preventive function of BBD in DEN-initiated HCC formation in rats and explored the possible mechanism. The results indicated that BBD could decrease the incidence of hepatocarcinogeneses induced by DEN in rats. Meanwhile, BBD could protect injured liver and decrease liver inflammatory infiltration. Moreover, we found that BBD could obviously inhibit ductal reaction and the expression of TLR4. *In vitro*, we used LPS to induce primary HPCs neoplastic transformation, and administrated with serum from BBD-treated rats. The results confirmed that BBD could inhibit primary HPCs neoplastic transformation via depressing the activation of TLR4. Furthermore, we illustrated the mechanism of BBD inhibited primary HPCs proliferation and neoplastic transformation by TLR4/Ras/ERK signaling pathway. Collectively, BBD may be a novel therapeutic choice for decreasing the incidence of HCC.

Babao Dan, a mixed powder of traditional Chinese medicine has been widely used as a complementary and alternative medicine to treat chronic liver diseases. Our previous study has demonstrated that BBD could ameliorate liver injury and the secretion of inflammatory cytokines. In fact, TLR4 is a classical inflammation-related signaling pathway common in tumor development, which is closely related to the tumor microenvironment, inflammatory response in the liver, and abnormal differentiation of liver precursor cells. The potential mechanism may be by inhibiting the expression of TLR4 pathway (23). In this study, we aimed to observe the effect of BBD on HCC formation and explored it possible mechanism.

Approximately 80% of HCC develop in fibrotic or cirrhotic livers, which occur in response to chronic liver injury caused by persistent inflammation (26). Thus, HCC also had been recognized as an inflammatory-associated tumor. In this study, we performed a hepatocarcinogenesis model in rats which was induced by intraperitoneal injections of DEN. Previous studies have demonstrated that DEN can arise chronic liver injure and induce the initiation of HCC. In present study, the results indicated that BBD could decrease the incidence of hepatocarcinogeneses induced by DEN in rats.

In chronic injured liver models, especially the proliferation or replication capacity of pre-existing functional hepatic cell to repairing is compromised, HPCs could be activated and chemotaxis to damaged hepatic parenchyma or bible duct areas, and then differentiate into adaptive hepatic parenchyma cells or bible duct epithelium cells to restore the injured liver (27). Chronic liver injury provides a microenvironment with many cytokines that favor tumorigenesis and tumor multiplication through cell activation, proliferation, migration and angiogenesis. The chronic liver injury results in a complex mixture of cytokines and growth factors secretion. IL-6 and TNF-α released by Kupffer cells stimulate the proliferation of HPCs, while interferon (IFN) enables HPCs to respond to mitogenic stimuli. Growth factors released by hepatic stellate cells from HPCs include EGF, HGF, TGF-α and TGF-β. So, cytokines released by Kupffer cells, hepatic stellate cells and HPCs themselves may act synergistically to control the proliferation of HPCs and remodeling of liver parenchyma (28, 29). Thus, decreasing the secretion of inflammatory factors could prevent the activation of HPCs. In the present study, the results indicated that BBD could decrease liver inflammatory infiltration and the secretion of inflammatory factors.

In histology reports, activation of HPCs in diseased liver fractions is described as ‘DR’ or ‘bile duct proliferation’ (30). DR expressing Sox-9 or CK19 may contain malignant degeneration of HPCs. The CK-positive HCC cells play a dominant role in directing aggressive behavior of tumor cell populations (31–33). Greater than 5% of cells in HCC expressing CK19 are associated with poor prognosis (8). The study of Sox-9 and CK-19 expression in the liver provides useful insights into the mechanisms of progenitor cell activity and tissue regeneration after liver injury. Many clinical studies have demonstrated that DR is related to HCC initiation and related to early recurrence (34, 35). In this study, the results demonstrated that DEN could induced DR and BBD could inhibit DR.

The activation of progenitor cells may directly promote hepatic carcinogenesis in liver regeneration (13–16). This hypothesis has been tested and confirmed in many rodent and human experiments. There is evidence showing that dysregulated HPCs have tumor initiation capacity *in vivo*, suggesting that HPCs may be involved in hepatocarcinogenesis (36, 37). In human chronic liver disease, particularly cirrhosis with chronic HBV or HCV infection, the proliferation of HPCs is directly correlated with disease severity, which suggested that the activation of this cellular compartment is associated with an increased risk of HCC development (38, 39). Many human HCC tumors contain a mixture of mature hepatocytes and an intermediate phenotype between HPCs and mature hepatocytes, similar to progenitor cells, suggesting HCC derived from progenitor cells (8, 40). In addition, these tumors exhibit the same gene expression profile as hepatocytes from HPCs. In retrospective studies, HCC tumors derived from HPCs showed a significantly poorer prognosis and a higher recurrence rate after surgical resection and liver transplantation (41, 42). Furthermore, in preclinical models, progenitor cells recruitment has been shown to contribute to hepatocellular carcinogenesis and might give rise to HCC as well as intrahepatic cholangiocarcinoma, supporting the idea that progenitor cells have an important role in the initiation and progression of liver cancers in some cases (43–46). These findings might also support the hypothesis of genuine liver cancer stem cells that might be derived from HPCs (44, 47).

Our previous study has demonstrated that HPCs could neoplastic transformation induced by LPS *in vitro*. TLR4, conferred by the lymphocyte antigen 96 also known as MD-2, can specially response to bacterial LPS, which was associated inflammation and fibrosis (48). Moreover, the potential mechanism may be through activation TLR4 pathway. LPS/TLR4 signaling can up-regulated the expression of Ras signaling pathway in HPCs, which can promote the proliferation and malignant transformation of HPCs (24). Meanwhile, Ras/ERK pathway plays a vital role in promoting cell proliferation, migration and invasion (49, 50). In this study, we also demonstrated primary HPCs possess tumor-initiating ability induced by LPS *in vitro*. Meanwhile, BBD could inhibit HPCs neoplastic transformation. Moreover, previous study suggested that TLR4 could facilitate tumor cells invasion and migration as a cancer stem cell marker in HCC (22). Subsequently, inhibiting the expression of TLR4 may be one of strategies to inhibit the initiate and progress of HCC. In this study, we found that BBD could effectively down-regulated the expression of TLR4/Ras/ERK.

## 5 Conclusions

In this study, we found that BBD could decrease the secretion of inflammatory factors, prevent the DR and the incidence of HCC in rats induced by DEN. The potential reason may be through inhibiting TLR4/Ras/ERK pathway. Collectively, BBD can be used as a potential adjuvant therapeutic choice to prevent HCC initiation in chronic injured liver. However, the monomer of BBD bioactive components needs be extracted and further research.

## Data availability statement

The original contributions presented in the study are included in the article/supplementary materials, further inquiries can be directed to the corresponding author/s.

## Ethics statement

The animal study was reviewed and approved by The Animal Ethics Committee of the Second Military Medical University, Shanghai, China.

## Author contributions

Z-PH, LZ and L-XW were responsible for the overall concept, design, and supervision of the study. LL and L-YZ performed the experiments and data analysis, as well as manuscript writing. W-TL, CZ, LG, RL, Q-DZ and N-PZ performed the experiments. All authors contributed to the article and approved the submitted version.
